# An algorithm for the management of traumatic abdominal wall hernia based on a 9‐year review

**DOI:** 10.1111/ans.18017

**Published:** 2022-09-01

**Authors:** Jessica Wong, Calvin Peng, Rose Shakerian, Brett Knowles, Ben Thomson, David J. Read

**Affiliations:** ^1^ The Trauma Service The Royal Melbourne Hospital Melbourne Victoria Australia

**Keywords:** abdominal wall hernia, blunt trauma, mesh repair, traumatic hernia

## Abstract

**Background:**

Traumatic abdominal wall hernia (TAWH) is a rare consequence of blunt abdominal trauma, usually in the setting of multitrauma, with little consensus or guidelines for management. We present a case series of patients with traumatic herniae over a 9‐year period and a suggested management algorithm.

**Method:**

Retrospective review of all patients with TAWH from 1st January 2011 to 31st December 2019 at a Level 1 adult Major Trauma Centre. Clinical presentation, surgical intervention and complications and recurrence were analysed.

**Results:**

Forty‐seven patients were found to have TAWH, 0.5% of all major trauma admissions. Thirty (63.8%) were repaired, 12 acutely, 11 semi‐acute and 7 delayed. All but 1 (fall>3 m) were transport associated, with a median Injury Severity Score (ISS) of 29. Follow‐up data for operative cases were available for all but one (97%). Seven (23.3%) cases had a recurrence, more common in the acute repair group (33.3%) compared to semi‐acute (18.2%), and elective group (14.3%).

**Conclusion:**

TAWH is a rare but potentially serious consequence of blunt abdominal trauma. This series has favoured earlier repair for anterior TAWH, or all those undergoing a laparotomy for other reasons, and elective repair for lumbar or lateral TAWH that do not require a laparotomy for other conditions. We present our preferred algorithm for management, accepting that there are many available strategies in this heterogeneous group of injuries. Loss of follow up and recurrence are a concern, and clinicians are encouraged to develop processes to ensure that TAWH are not a ‘forgotten hernia’.

## Graphical abstract

Traumatic abdominal wall hernia is a rare but potentially serious consequence of blunt abdominal trauma. We present a case series of patients with traumatic herniae over a 9‐year period and a suggested management algorithm.

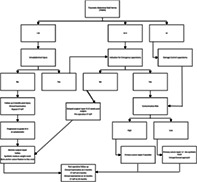

## Introduction

Traumatic abdominal wall hernia (TAWH) refers to disruption in the abdominal wall musculature following injury.^1^ Blunt TAWH often result from deceleration injury with avulsion of abdominal wall muscles from bony attachments,^2^ which may not be familiar to many elective hernia surgeons. These injuries are rare, comprising less than 1% of blunt trauma admissions^3^, ^4^ usually seen in the context of the multi‐injured patient,^5^ and are associated with an intraabdominal injury requiring laparotomy in approximately a third.^6^

TAWH are a heterogeneous group of injuries, with little consensus on management and available evidence limited to a few single centre case series^3^, ^4^, ^7^ and one multicentre retrospective review.^6^ We hypothesize that TAWH rarely accounts for immediate complications and best approached in a delayed elective manner, using a synthetic mesh. We present a 9‐year series from a Level 1 Major Trauma Centre and offer a suggested management algorithm.

## Methods

This study is a retrospective analysis of prospectively collected dataset from the Royal Melbourne Hospital (RMH) trauma registry and contains patients who presented from 1st January 2011 to 31st December 2019 with TAWH.

The RMH is one of two adult, Level 1 Major Trauma Centres in Victoria, Australia. RMH manages approximately 1200 major trauma admissions *per annum*. The Trauma Registry includes all patients who meet the Victorian Major Trauma Criteria.^8^ The Major Trauma criteria is defined as those with an Injury severity score (ISS) > 12, or an intensive care unit (ICU) admission >24 h requiring mechanical ventilation, or urgent surgery for intracranial, intrathoracic or intraabdominal injury, or fixation of pelvic or spinal fractures, death after injury, or a burn of >20% total surface area.^8^

The RMH Trauma Registry was searched for free text ‘hernia’ and AIS codes (510 100.2, 510 800.1, 510 802.1, 510 804.2, and 510 806.3). Adult patients meeting Major Trauma Criteria with a Grade 3 TAWH or above^1^ (Table 1) admitted between 1st January 2011 to 31st December 2019 to the RMH were included in the study. Patients' medical records and imaging were reviewed by authors to confirm fulfilment of inclusion criteria. All patients with TAWH, regardless of whether the herniae were repaired, were included in the study. Patients with herniae following penetrating injury or those who died in the Emergency Department were excluded.

**Table 1:** Site, grade, and timing of repair of TAWH

Site	Definition
Anterior	Rectus abdominus disruption
Lateral	External or internal obliques or transversus abdominus
Lumbar	Superior and inferior lumbar triangle
Grade	Definition(1)
1	Subcutaneous tissue contusion
2	Abdominal wall muscle haematoma
3	Single abdominal wall muscle disruption
4	Complete abdominal wall muscle disruption
5	Complete abdominal wall muscle disruption with herniation of abdominal contents
6	Complete abdominal wall disruption with evisceration
Timing of repair	Definition
Acute	Repair during index trauma laparotomy
Semi‐acute	Repair during subsequent laparotomy within the same hospital admission
Elective	Performed during separate admission

**Fig. 1:**
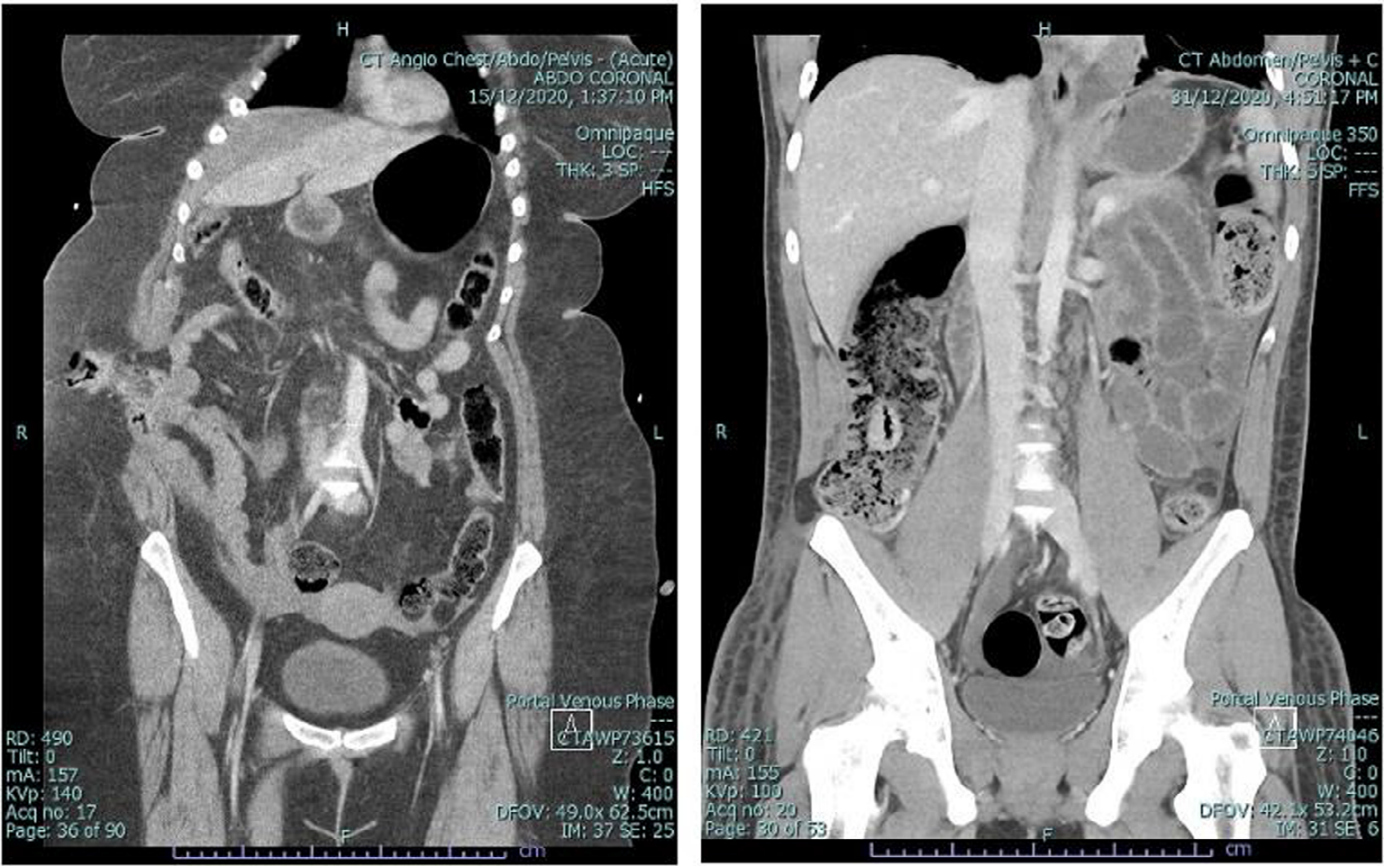
TAWH on CT.

TAWH were categorized into site, grade and timing of repair (Table 1) (Fig. 1).^1^ Patients without evidence of follow up in the RMH medical records were contacted directly to ascertain if they had surgery or had complications managed elsewhere, via a structured interview. Variables included demographics, mechanism of injury, clinical and radiological characteristics of the TAWH, concomitant injuries, surgical intervention, post‐operative morbidity, and any in hospital mortality.

Continuous variables were summarized as median and interquartile range (IQR) and categorical variables as frequencies and percentages. Patients' groups were compared using Mann–Whitney test for continuous variables and Chi‐square test or Fischer's exact test for categorical variables. A *P*‐value of <0.05 was considered significant. All statistical analyses were performed using Statistical Package for Social Sciences (SPSS). This project was approved by the Melbourne Health Ethics and Research Governance (QA2020064).

## Results

In total, 8930 patients with major trauma were identified during the study period. Of the 58 that were identified within the Trauma Registry, 11 were excluded as they did not have TAWH. 47 (0.5%) patients were confirmed to be eligible and had TAWH secondary to a blunt injury (Table 2). Two thirds of the 47 patients were admitted in the second half of the study (1st July 2015–31st December 2019). There were three inpatient deaths (6%) secondary to unsurvivable extra‐abdominal injuries whereby hernia management was not appropriate.

**Table 2:** Demographics of patients with traumatic abdominal wall hernia

Demographics		
Age (years)	Median, IQR	46 (31.6–60.5)
Gender	Female, *n* (%)	26 (55.3)
Mechanism, *n* (%)	Motor Vehicle Collision	37 (78.7)
	Motorcycle Collision	7 (14.9)
	Pedestrian	2 (4.3)
	High fall	1 (2.1)
Associated extra‐abdominal injuries^†^, *n* (%)	Head	12 (25.5)
	Chest	27 (57.4)
	Spine	38 (80.9)
	Pelvis	8 (17.0)
	Limbs	25 (53.2)
ISS	Median, (IQR)	29 (20.5–37.5)
ICU admission	Yes (%)	18 (38.3)
LOS ICU	Median (IQR)	3.5 (0.7–6.4)
LOS total	Median (IQR)	10.5 (3.1–17.9)
Mortality, *n* (%)		3 (6.4)
Site of hernia	Grade of hernia	Number (%)
Anterior hernia	3	1 (2.1)
	4	5 (10.6)
	5	0 (0)
	6	1 (2.1)
Lateral hernia	3	1 (2.1)
	4	11 (23.4)
	5	9^‡^ (19.1)
Lumbar hernia	4	13 (27.7)
	5	6 (12.8)

^†^ Multiple injuries possible.

^‡^ includes one case with synchronous lateral and anterior hernia.

Thirty‐one (66.0%) patients underwent a trauma laparotomy during their admission. The intra‐abdominal injuries included small bowel 15 (32%), colonic 13 (28%), mesenteric 5 (11%), diaphragmatic 3 (6%), spleen 4 (8%), ovary 1(2%), renal tract 1 (2%), and vascular 1 (2%) (aortic and SMA).

### Management

Thirty (63.8%) of the 47 patients underwent surgical repair of their TAWH (Table 3). Twelve (40.0%) were repaired acutely, 11 semi‐acutely (36.7%) and 7 electively (23.3%) (Table 4). The open approach was utilized in 29 patients (96.7%) and mesh utilized in 13 (43.3%). The median length of time from discharge to elective repair was 133 days (IQR 85–291).

**Table 3:** Demographics of patients with operative versus non‐operative TAWH

		Operative	Non‐operative	*P*‐value
		*n* = 30	*n* = 17	
Age	Median, (IQR)	44.1 (29.2–59.1)	53.2 (41.1–65.4)	0.39
Gender	Female (*n*, %)	16 (53.3)	10 (58.8)	0.72
ISS	Median, (IQR)	24 (15.5–32.5)	30 (25–35)	**0.03**
Mechanism (*n*, %)	Motor vehicle collision	23 (76.7)	14 (82.4)	0.47
	Motorcycle collision	5 (16.7)	2 (11.8)	
	Pedestrian	2 (6.7)	0 (0)	
	Fall	0 (0)	1 (5.9)	
LOS	Median, (IQR)	11.1 (5.5–16.7)	13.3 (21.8)	0.87
ICU Admission	Yes (*n*, %)	5 (16.7)	13 (76.5)	**<0.001**
ICU LOS	Median, (IQR)	7 (2–12)	3 (4)	0.29
Trauma Laparotomies	Yes (*n*, %)	20 (66.7)	11 (64.7)	1.00
Side of hernia^†^	Right	20	10	0.14
	Left	4^‡^	6	
Site	Lateral	11^‡^	10	0.27
	Lumbar	13	6	
	Anterior	6	1	
Grade of hernia	Grade 3	0	2	0.19
	Grade 4	18	11	
	Grade 5	11	4	
	Grade 6	1	0	

^†^ Excludes 7 anterior herniae.

^‡^ Includes one case with synchronous lateral and anterior hernia.

**Table 4:** Surgical management and recurrences

Surgical management & recurrences				
			Number (%)	Recurrence *n* (%)
Timing	Acute		12 (40.0)	4 (33.3)
	Semi acute		11 (36.7)	2 (18.2)
	Delayed		7 (23.3)	1 (14.3)
Approach	Open		29 (96.7)	7 (24.1)
	Laparoscopic		1 (3.3)	0 (0)
			Number (%)	Recurrence *n* (%)
Suture repair			17 (56.7)	6 (35.3)
Mesh (*n* = 13)	Bio‐A®		6 (20.0)	
	Synthetic		4 (13.3)	
	Composite		3 (10.0)	1 (7.7)
Recurrences				
Reported as	Timing	Site	Repair	Management
Clinician confirmed	Acute	Lateral	Direct suture	Open repair with Bio‐A® mesh^†^
Self‐reported	Acute	Lateral	Direct suture	conservative
Self‐reported	Acute	Lumbar	Direct suture	conservative
Self‐reported	Acute	Lateral	Direct suture	conservative
Clinician confirmed	Semi‐acute	Lateral	Direct suture	Delayed open mesh repair^‡^
Self‐reported	Semi‐acute	Lateral and anterior	Direct suture	conservative
Clinician confirmed	Delayed	Lumbar	Mesh	Open repair, mitec screws^§^

^†^ Patient A.

^‡^ Patient B.

^§^ Patient C had two recurrences.

Of the 17 (36.2%) did not have their herniae repaired, 1 patient's medical record was not recovered, and 3 patients died. Eight had lateral hernia, 4 had lumbar hernia and 1 had partial defect in left rectus abdominus without complete muscle disruption. Eight of these 13 patients had trauma laparotomies for other injuries. These were colon,^4^ small bowel,^4^ spleen,^1^ diaphragm,^1^ mesentery,^1^ and vascular injuries.^1^

There were no statistically significant differences in the age, gender, mechanism, hernia location, trauma laparotomy rates, LOS, and ICU LOS between the operative and the non‐operative group (Table 3). However, the non‐operative group had a higher ISS (median ISS 30 versus 24, *P* = 0.03) and were more likely to be admitted to ICU than the TAWH‐operated group (76.5% versus 16.7% *P* < 0.001).

### Complications and recurrences

Median follow up of 12 months (IQR 4–20) was available for 29 of 30 operative and 9 of 13 non operative cases. The recurrence rate was 7 of 30 patients (23.3%). Three of the 30 repairs had a documented recurrence and repair at our institution, and on phone contact a further four recurrences were reported. Follow up appointments were offered for these patients.

Patient A had an acute suture repair of a right lateral hernia and presented 49 days later with an irreducible hernia containing bowel. This was repaired open with Bio‐A® mesh. Patient B had a semi‐acute suture repair of a right lateral hernia post a trauma laparotomy, splenectomy, and small bowel resection. Patient B developed anastomotic leak, intraabdominal sepsis, enterocutaneous fistula and an early recurrence 10 days post repair. Patient B underwent an ileocolic resection, excision of fistula and formation of ileostomy, multiple laparostomies before abdominal wall closure. Fourteen months later, patient B had an open sublay prolene mesh repair. Patient C had two recurrences after elective repair of a left lumbar hernia. The first recurrence was a month later when the composite mesh had come off the iliac crest. The second recurrence was 5 months after repair when the composite mesh had fractured. The third repair was performed with a prolene repair in sublay fashion.

Recurrence rate by timing of repair is shown in Table 4, which demonstrates that a trend of the later the repair, the lower the recurrence rate, though the low numbers precluded meaningful statistical analysis. There was also a trend of a higher recurrence rate in suture repair (35.3%) compared to mesh repair (7.7%), although it was not statistically significant (*P* = 0.15) (Table 4). Other complications included three seromas (10%) requiring interventional radiological drainage.

## Discussion

This series of 47 patients with TAWH had a similar incidence (0.5%), recurrence rate (23.3%) and wound complication (10%) compared to the literature.^3^, ^4^, ^6^, ^9^, ^10^ This series' recurrence rate is toward the higher end of the 7.3%–26.7% range described in the literature, but has the advantage of diligent follow up of 97% of operative cases. The rarity of this condition has meant there is only low number observational data on which to base management. Accepting this constraint, we present our preferred way of managing TAWH, which has evolved over time, acknowledging that there are other management strategies, particularly around the indications, timing, and method of repair.

TAWH is a deceleration injury, and it is often associated with seat‐belt sign. Firstly, the diagnosis and treatment of TAWH are often low priorities in the multitrauma patient. In our own series, there was almost twice the number of TAWH diagnosed in the latter half of the study (1st July 2015–31st December 2019) compared to the first half (1st January 2011–30th June 2015) (31 versus 16). We suggest including TAWH in the daily radiology review checklist of all admission scans performed in the last 24 h. Increased awareness is the first step in reducing missed injury or delayed diagnosis.

Secondly, TAWH is associated with intra‐abdominal pathology. 66% of our patients needed a trauma laparotomy for either bleeding or bowel injury. We recommend TAWH on its own is not an indication for laparotomy, unless it is a grade 6 injury. However, clinicians are encouraged to have a high index of suspicion of intraabdominal injury in patients with TAWH and operate if there is clinical deterioration.

If a hernia is encountered at the time of index trauma laparotomy, the patients are usually physiologically deranged, repair requires suturing freshly torn muscle, and the space is often contaminated limiting the use of mesh. Our non‐operative patients had a significantly higher ISS (*P* = 0.03) and were more likely to be admitted to ICU (*P* < 0.001). Some authors recommend immediate repair due to the perceived increased risk of strangulation.^11^, ^12^ However, more recent studies have shown that patients may be safely observed and have their TAWH repaired electively.^3^, ^13^, ^14^ Netto showed that 64.7% of patients did not develop symptoms in the follow up period (range 1–16 month).^14^ In the multicentre series from Harrell *et al*. however, the vast majority (90%) were repaired in the index hospital admission, not that dissimilar to the rate in our current series (77%).^6^

Over time, our preference has drifted toward delayed repair if feasible, aiming for 2–3 months post injury, when the patient is over the acute phase of their care. There is a divided opinion about the effect of the timing of repair on recurrence rate. Netto and Honaker reported increased recurrence rates in patients undergoing an acute repair,^14^, ^15^ which is supported by the results of this study. However, the largest series in the literature showed no difference in recurrence in early (defined as during index admission, 11.5%), and late repair (after discharge, 15.8%, *P* = 0.869).^6^ It should be noted that all of these were observational studies without adjustment for confounding factors.

There are times that delayed repair is impractical. These include evisceration in a Grade 6 injury, concomitant intraabdominal injury requiring laparotomy, or rarely, immediate enteric strangulation from the TAWH. In these situations, we prefer direct repair if feasible with biologic or Bio‐A® mesh. If it is not possible to directly repair the hernia, we follow these patients closely. Finally, as reported by Netto,^14^ we also believe that anterior TAWH have a higher chance of concomitant intrabdominal injury and subsequent strangulation, so we prefer early repair for these TAWH.

**Fig. 2:**
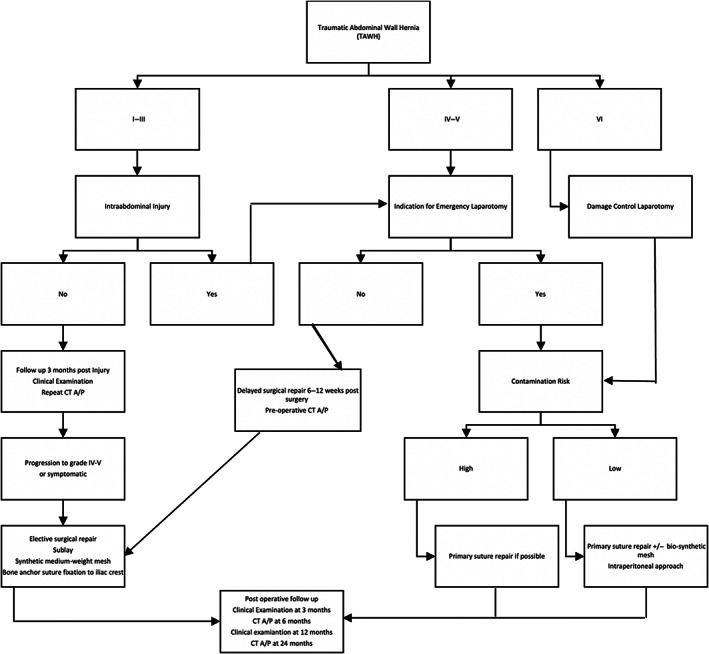
Algorithm of management of TAWH.

Based upon our Institution's experience, we present an algorithm for management and follow up of TAWH (Fig. 2). A previous algorithm has been published, reasonably taking into contamination and hernia size, but also relying on a self‐assessed ‘surgeon's skill’ which is open to interpretation and ISS, which is calculated retrospectively after all injuries have been identified and therefore not a decision‐making tool to be used at point of care.^16^ Grade 1 to 2 TAWH are soft tissue injuries, and in our opinion not true hernia. If encountered at the time of laparotomy, our recommendation is to avoid extensive debridement in the acute setting, being aware that local complications such as infected seroma or skin necrosis can develop.

We suggest repairing anterior abdominal hernia if feasible in an acute or semi‐acute setting, after adequate debridement of contaminated or devitalized tissue. This is especially pertinent in patients who also had damage control laparotomy. For lateral and lumbar herniae, we try to postpone repair till the elective setting if feasible, which based on our experience, is associated with a lower recurrence rate, albeit not significant. If a lateral or posterior TAWH is discovered at trauma laparotomy for other indications, then the preferred options are to leave the defect alone if there is no herniation, noting that direct repair of freshly torn muscle in a physiologically compromised patient seems to have great potential for recurrence, and enteric injury may limit the utilization of mesh. If the defect requires repair due to herniation, the direct or mesh repair are option with subsequent close follow up.

In terms of technique for delayed repair, described options for operative repair include direct suture repair, repair with either synthetic, composite or biological mesh, which can be performed by open or laparoscopic approach.^16^ As many of these injuries involve muscle torn off bony prominences, our preferred method of repair is delayed with synthetic medium weight mesh, often utilizing bone anchoring screws to the iliac crest.^17^ The mesh is ideally placed in the preperitoneal plane, with large overlap into psoas posteriorly and inguinal and femoral canals as described in transversus abdominis muscle (TAR) release plane. A delayed repair gives time for scar tissue to form, which we feel is more likely to hold sutures than freshly damaged muscle. We had a low rate of utilization of laparoscopy in this series. Although laparoscopy is certainly an option, we prefer a direct open approach, bypassing any potential intrabdominal adhesions, and giving more repair options such as bone anchoring sutures, and direct suture repair.

Finally, we have recently formalized clinical and radiological follow up of both Grade 3 and above TAWH injuries and postoperative patients, given the relatively high risk of recurrence and experienced difficulty in follow up.

### Limitations

This is a single centre series and therefore the results may have limited generalisability. Its retrospective nature means there are potentially other factors that might have impacted the decision to operate. The hypothesis regarding evidence to support delayed and mesh repair could not be substantiated due to the limited number in this study. It is stressed that the management algorithm offered, represents the opinions of a few surgeons, and is not exclusive of other management options. Although the majority of cases were followed up, many were assessed via telephone, and hence we are relying on the patient's assessment of recurrence and complications, which could be inaccurate. Finally, we discovered 14 different ICD codes in usage for this injury, and it is possible with such variability that other cases could have been missed.

## Conclusion

Traumatic Abdominal Wall Herniae are an uncommon but potentially increasingly recognized consequence of the severely injured patient. These injuries' rarity, heterogeneity, lower immediate priority, and lack of consistent coding have been impediments to developing management strategies. This series has favoured earlier repair for anterior TAWH, or all those undergoing a laparotomy for other reasons, and elective repair for lumbar or lateral TAWH that do not require a laparotomy for other conditions. In general, we favour planned elective repair due to the perceived higher risk of recurrence in early repair in physiologically unwell trauma patients. In those undergoing a trauma laparotomy, we advocate minimalistic repair, direct closure and close follow up due to the risk of recurrence. Loss of follow up and recurrence are a concern, and clinicians are encouraged to develop processes to ensure that TAWH are not a “forgotten hernia”.

## Author contributions

**Jessica Wong:** Data curation; formal analysis; methodology; project administration; writing – original draft; writing – review and editing. **Calvin Peng:** Data curation; formal analysis; project administration; writing – review and editing. **Rose Shakerian:** Conceptualization; writing – review and editing. **Brett Knowles:** Conceptualization; writing – review and editing. **Ben Thomson:** Conceptualization; writing – review and editing. **David J. Read:** Conceptualization; formal analysis; methodology; supervision; writing – review and editing.

## Conflict of interest

None declared.
